# 老年肺癌护理中国专家共识（2022版）

**DOI:** 10.3779/j.issn.1009-3419.2023.102.05

**Published:** 2023-03-20

**Authors:** Lung Cancer Specialty Committee of Chinese Elderly Health Care Association, Enhanced Recovery after Surgery Specialty Committee of Tianjin Medical and Health Association

**Keywords:** 老年, 肺肿瘤, 护理, 共识, Elderly, Lung neoplasms, Nursing, Consensus

## Abstract

鉴于老年肺癌患者治疗的前瞻性研究不多，我们在借鉴《老年肺部手术围手术期加速康复护理专家共识》的同时，仍需关注放化疗及免疫靶向治疗的老年肺癌患者的护理管理。为此，中国老年保健协会肺癌专业委员会组织全国部分胸科医护专家，依据当前国内外最新研究进展及最佳的临床证据，牵头编写了《老年肺癌护理中国专家共识（2022版）》。通过检索国内外相关文献并结合我国临床实际情况，以循证医学为基础，以问题为导向，针对老年肺癌患者不同的治疗手段，进一步规范评估工具的应用、指导落实临床症状观察及护理措施、关注老年患者各种高危风险因素的预防，以多学科合作为模式，整体化护理为内涵，制定本共识，以期使老年肺癌患者治疗和护理实践更为规范，更具针对性，从而降低并发症的发生，为指导未来临床研究提供参考依据。

## Abstract (en)

In view of the limited data from the current prospective studies on the treatment of elderly patients with lung cancer, while drawing on The expert consensus of accelerated rehabilitation nursing in peri-operative period of lung operation in the elderly, the nursing care of the aged patients with lung cancer still needs to be concerned about radiotherapy, chemotherapy and immuno-targeted therapy. To this end, Lung Cancer Specialty Committee of Chinese Elderly Health Care Association organized a national team of thoracic medical and nursing experts, based on the latest research progress and the best clinical evidence at home and abroad, took the lead in preparing Consensus of Chinese experts on nursing of lung cancer in the elderly (2022 edition). Based on evidence-based medicine (EBM) and problem-oriented medicine, the author searched the relevant literatures at home and abroad and combined with the clinical situation in our country, aimed at the different treatment methods of the aged patients with lung cancer, to further standardize the application of assessment tools, guide the implementation of clinical symptoms observation and nursing measures, pay attention to the prevention of various high-risk factors of elderly patients, to multidisciplinary cooperation as a model, holistic nursing as the connotation, develop this consensus. In order to make the treatment and nursing practice of senile lung cancer patients more standardized, more targeted, so as to reduce the occurrence of complications, and also provide references and guidances for related clinical research.

肺癌是中国最常见的高发恶性肿瘤，其死亡率和发病率均位于恶性肿瘤的第一位。根据国家癌症中心2022年公布的数据^[1]^可见，中国癌症发病排第一位的是肺癌，发病率为59.89/10万，死亡率为47.51/10万。因此肺癌已成为中国乃至全球癌症死亡最常见的原因之一^[2]^。肺癌的发病率与年龄成正比，老龄化日渐严重，老年人数量不断增加，肺癌发生率也不断增加。从2005年-2014年，中国老年肺癌患者比例从41.2%升至56.2%^[3]^。

随着医学水平的不断提高，肺癌的治疗手段也在不断丰富，除手术、放疗、化疗以外，免疫与靶向治疗也让多数肺癌患者看到了疗效^[4]^。老年患者生理机能退化，常常伴有多种合并症、衰弱等身体因素，尤其是老年肺癌患者肺功能降低，对各种治疗的不良反应耐受性较差，如何降低围手术期风险、降低术后及放化疗、免疫与靶向治疗后并发症的发生，维持治疗后各器官功能状态，都需要护理工作者做好密切的临床观察与护理。

目前关于老年肺癌患者治疗的相关研究内容有限，在借鉴《老年肺部手术围手术期加速康复护理专家共识》的同时，仍需关注接受放化疗及免疫靶向治疗的老年肺癌患者的护理。为此，中国老年保健协会肺癌专业委员会组织全国部分胸科医护专家，依据当前国内外最新研究进展及最佳的临床证据，牵头编写了《老年肺癌护理中国专家共识（2022版）》。通过检索国内外相关文献并结合我国临床实际情况，以循证医学为基础，以问题为导向，对于老年肺癌患者不同的治疗手段，进一步规范评估工具的应用、指导落实临床症状观察及护理措施、关注老年患者各种高危风险因素的预防，以多学科合作为模式，整体化护理为内涵，制定本共识，以期使老年肺癌患者治疗和护理实践更为规范、更具针对性，从而降低并发症的发生，也为未来临床研究提供了参考依据和指导意见。

本共识包括共性问题及外科手术、化疗、放疗、免疫和靶向治疗五个各论部分，总论汇总了老年肺癌患者护理的共性问题，后者分别就不同治疗手段中老年肺癌患者的具体问题展开讨论。总论部分已涉及的共性问题，各论部分从简。

## 老年肺癌患者的共性问题

**问题1：老年肺癌患者存在哪些安全风险**

**共识推荐：老年肺癌患者需做全面的风险评估及管理，以预判患者存在的安全隐患，做好治疗期间的安全管理。安全管理建议包括跌倒/坠床、非计划拔管（unplanned extubation, UEX）、皮肤压力性损伤、静脉血栓的风险评估及管理。**

**（1）跌倒/坠床风险评估及预防**

**证据：**疾病的消耗和治疗后的不良反应导致老年肺癌患者全身疲乏，容易发生跌倒/坠床^[5]^。多数患者和家属对预防跌倒/坠床的认知不够，尤其是一些低龄老人，依从性差、拒绝陪护，因活动不当容易造成跌倒/坠床^[6]^。

**观察护理：**老年患者发生跌倒/坠床的危险因素包括年龄、跌倒史、步态、疾病因素、认知功能、药物的使用、环境等。目前已知的跌倒危险因素超过400个^[7]^。通过跌倒风险评估，可快速识别跌倒高危患者，及时采取针对性的措施。常用的评估量表有Morse跌倒风险评估量表、Hendrich II跌倒风险评估量表、约翰霍普金斯评估量表、STRATIFY评估量表等^[8⇓⇓-11]^，如表1所示。预防措施：建议家属陪伴，患者伴有较为剧烈的咳嗽时，应防止晕厥。应保证环境安全和设施齐全，如病房应光线充足，过道通畅无障碍，设置夜灯，厕所、过道设置扶手，湿滑地面设置警示标志。患者改变体位时应缓慢，下床活动时应着长短适当的衣物、穿防滑鞋。行走不便者，应使用合适的辅助器材。做好排泄相关跌倒预防。合理使用床挡，必要时使用保护性约束。

**表1:** 老年肺癌患者风险评估量表汇总

序号	项目	表格
1	跌倒坠床风险评估	Morse跌倒风险评估量表、Hendrich II跌倒风险评估量表、约翰霍普金斯跌倒风险评估量表、 STRATIFY跌倒评估量表等
2	压力性损伤的风险评估	Norton压疮评估量表
3	静脉血栓风险评估	Caprini风险评估量表
4	营养风险筛查	NRS 2002量表
5	护理人员灵性照顾观念	灵性照顾认知量表（SCGS汉化版）、灵性与灵性照顾测评量表（SSCRS汉化版）
6	护理人员灵性照顾能力	灵性照顾能力量表（SCCS汉化版）
7	患者灵性照顾需求	护理人员灵性照顾需求量表（NSTS汉化版）
8	谵妄评估	意识模糊评估法
9	化疗导致的恶心呕吐的评估	Dranitsaris风险评分系统
10	癌因性疲乏评估	简易疲乏量表、癌症疲乏量表等

NRS：营养风险筛查；SCGS：灵性照顾认知量表；SSCRS：灵性与灵性照顾测评量表；SCCS：灵性照顾能力量表；NSTS：护理人员灵性照顾需求量表。

**（2）UEX风险评估及预防**

**证据：**住院患者有意造成或任何意外造成的拔管都属于UEX，即在医护人员非诊疗计划范畴内的拔管^[12,13]^。UEX会增加患者痛苦、造成身体损伤甚至危及生命健康，且易引发医患矛盾^[13,14]^。对患者而言，降低UEX、提高管路安全是护理安全管理的重点环节，也是中国患者安全管理的目标之一。提高管路安全的首要工作是建立管路安全管理制度以及建立风险评估的流程^[14,15]^。

**观察护理：**老年人UEX的常见因素包括躁动、谵妄、不配合、麻醉未醒、紧张害怕、舒适度差及无法与医护人员有效沟通等。UEX风险最高且后果严重的导管以气管插管、胸腔闭式引流管为主。目前关于UEX风险尚未有一个全面、统一的评价工具。预防措施包括：妥善有效固定导管；做好患者及家属的充分告知，明确留置导管的重要性；充分评估管道固定情况，安全操作；意识不清或烦躁谵妄患者必要时使用保护性约束；医护沟通，尽早拔管。

**（3）压力性损伤的风险评估**

**证据：**老年肺癌患者的皮肤弹性降低，削弱了皮肤的防御能力，局部组织一旦受压极易造成血运不畅^[16]^；老年肺癌患者的整体抵抗力下降，自理能力受限，行动不便，居家或疗养期间长期卧床，进一步增加了发生压力性损伤的风险^[17]^。压力性损伤除了会引起局部组织受压缺氧缺血，还会引发整个机体的营养不良，导致机体软组织溃烂，甚至坏死，给患者及其家庭带来负担^[18]^。存在压力性损伤的老年肺癌患者在肺癌治疗及护理期间存在较多风险，护理人员应加强此类患者的护理管理。

**观察护理：**老年人压力性损伤的风险因素包括年龄、消瘦或肥胖、大量出汗、大小便失禁、合并糖尿病、营养不良、长期卧床等。推荐使用Norton量表进行老年患者压力性损伤风险评估^[19]^。通过评估，快速识别压力性损伤高危患者，及时采取针对性的措施。

**（4）静脉血栓风险评估及预防**

**证据：**老年肺癌患者存在多种静脉血栓栓塞症（venous thromboembolism, VTE）危险因素，一旦发生肺栓塞，后果严重、死亡率高。老年人血管壁弹性纤维减少，血管增厚变硬，血液凝固性高，加之手术创伤引起的炎症反应及术后初期的绝对卧床等，以上因素均可导致老年患者血栓形成，下肢血栓脱落可造成肺栓塞。

**观察护理：**患者入院时推荐使用Caprini风险评估量表对患者进行常规静脉血栓风险评估^[20,21]^，并根据评估结果进行分级预防。

术后血栓预防：①早期活动：患者麻醉苏醒后可在床上进行四肢的主动运动，术后第1天，可在家属或医护人员的协助下进行床旁活动1 h-2 h，术后第2天起至出院时每日活动4 h-6 h^[22]^。我国老年患者受传统观念的影响，认为手术后应增加卧床休息时间，医护人员应向患者解释早期下床活动的优势以及长期卧床的不利影响，鼓励患者完成每日活动目标，根据患者自身情况，循序渐进地增加活动量；②指导患者保持大便通畅，避免因便秘诱发肺动脉栓塞（pulmonary embolism, PE）；③密切观察病情变化，如突然出现的呼吸困难、胸痛、胸闷、咳嗽、血痰、大汗、心率/呼吸增快，甚至晕厥休克，高度警惕发生PE的可能，此时患者应立即平卧；④避免下肢静脉或股静脉穿刺，特别是下肢反复穿刺；⑤下肢深静脉血栓是导致PE的重要因素，密切观察卧床老年患者双下肢肤色、温度、肿胀程度及感觉，必要时同时测量双下肢同一平面的周径；⑥使用抗凝溶栓药物期间，注意观察患者有无咳嗽、呼吸困难等PE症状。

**问题2：老年肺癌患者营养风险筛查（nutritional risk screening, NRS）及营养不良的干预**

**共识推荐：患者入院时应用NRS 2002量表对患者进行常规NRS^[23]^，若得分≥3分，则需通知主管医生并请营养科会诊，根据会诊意见对患者进行营养干预。欧洲营养与代谢协会推荐的指标评估如下：（1）在近6个月内体重骤降10%-15%或更多；（2）进食量<推荐摄入量的60%，持续超过10 d；（3）体质指数低于18.5 kg/m^2^；（4）除肝肾功能异常者，清蛋白<30 g/L。**

**证据：**相关研究^[24]^显示，肺癌患者大多存在营养不良等症状，老年人本身体质弱，机体代谢较年轻人缓慢，所以老年肺癌患者的营养状态会更差。专家推荐膳食管理联合营养管理^[25]^，依据老年肺癌患者的基本情况，及时补充三大必需营养物质，促进患者早日恢复。

**观察护理：**根据患者个体情况设立营养目标，优先采用口服营养补剂（oral nutritional supplement, ONS）或肠内营养^[26]^。可经口进食的患者，膳食管理联合营养管理可有效改善老年肿瘤患者营养状态，通过对患者营养状态进行评估，结合实验室指标制定膳食计划，选择高热量、优质蛋白质饮食[热量25 kcal/（kg·d）-30 kcal/（kg·d）、蛋白质1.0 g/（kg·d）-1.2 g/（kg·d）]。

**问题3：老年肺癌患者灵性照顾的重要性及方法**

**共识推荐：老年肺癌患者因疾病预后较差以及治疗不良反应较多等问题，易产生焦虑、无助等负性情绪，逐渐丢失人生意义及价值感，因此推荐对老年肺癌患者进行灵性照顾。推荐使用灵性照顾认知量表（SCGS汉化版）[27]、灵性与灵性照顾测评量表（SSCRS汉化版）[28]对护理人员灵性照顾观念进行测评，使用灵性照顾能力量表（SCCS汉化版）[29]对护理人员灵性照顾的能力进行测评，以协助临床护理管理者来评估护理人员对灵性和灵性照顾的理解，以制定更有效的管理策略，帮助护理人员提高灵性照顾能力。使用护理人员灵性照顾需求量表（NSTS汉化版）[30]对患者灵性照顾的需求进行测评，了解患者灵性照顾的需求[31]。推荐使用叙事护理的方法对患者进行灵性照顾。**

**证据：**灵性影响着一个人的健康^[32]^，也是整体护理的重要内容之一^[33]^。灵性照顾可有效提升患者的机体功能，减少悲痛、抑郁等不良情绪，同时促进幸福感^[34]^。在慢性疾病或癌症的护理工作中，人们越来越认识到灵性健康的重要性^[35]^，安宁疗护的临终患者尤其重视灵性健康，晚期癌症患者也渴望得到灵性照顾^[36]^。灵性照顾的主要提供者来自护理人员，其灵性照顾时的态度对实施有直接影响^[37]^。

**观察护理：**灵性照顾是在灵性护理价值观的引导下护理人员的态度与行为^[38]^，认可人类尊严、安宁、慈悲、善良、照顾自我及他人等观点。在疾病治疗过程中帮助患者寻求自我价值、感悟生命意义、给予爱与宽恕、感受信念与信任、消除对死亡的恐惧，缓解不确定和不舒适感，最终恢复内心的宁静^[39]^。灵性照顾的概念包含了四个相互关联的要素：一是作为灵性照顾临床实践的实施者即护理人员及其灵性能力；二是作为被照顾者即患者的灵性需求；三是护理人员针对患者灵性需求的有目的、有针对性的回应；四是以灵性护理技能为实践基础，旨在帮助患者缓解痛苦、提升灵性健康水平。总之，护理人员通过评估患者的灵性需求，运用专业的临床护理知识与技能，协助或回应患者寻求希望与宁静、缓解痛苦、走出困境。

老年肺癌患者灵性需求偏低，主要的影响因素包括病程、经济条件、宗教信仰和文化水平等。护理人员首先需充分了解患者的灵性需求，尤其在宗教信仰等方面，另外不断提升自身能力，提高灵性照顾的意识，熟练掌握评估的方法，正确对待灵性问题，为患者提供更精准的灵性照顾，提升生活质量。

叙事护理是一种新型的心理护理模式，它以叙事医学理论为基础，结合了后现代心理的叙事治疗的方法、理念和临床护理实践。护理人员在倾听患者故事的过程中，重构其疾病故事的意义，建立良好的护患关系，发现护理切入点，对其实施护理干预，目的是消除患者内心苦闷或困惑^[40,41]^。叙事护理广泛应用于肿瘤患者的护理干预中，研究^[42]^显示临床效果较好。

## 老年肺癌患者围术期护理

**问题1：老年肺癌患者气道并发症风险评估及预防**

**共识推荐：术前应常规对老年患者进行肺功能及气道风险评估，包括：年龄>70岁、吸烟指数>400支/年、哮喘、气道高反应性（airway hyper reactivity, AHR）、慢性阻塞性肺疾病（chronic obstructive pulmonary disease, COPD）、肥胖或体表面积（body surface area, BSA）>1.68 m2、肺功能较差、呼气峰值流量（peak expiratory flow, PEF）<300 L/min、营养代谢紊乱、既往放化疗史、手术史及致病性气道定植菌等[43]。根据评估结果选择合适的肺康复方式对患者进行指导。**

**证据：**术前合并高危因素的老年肺癌患者建议进行为期至少7天的肺康复训练，有助于改善术前的心肺功能，有效降低术后肺部并发症，促进患者康复^[44]^。

**观察护理：**（1）戒烟：研究^[45,46]^报道显示，吸烟指数>400支/年的患者是开胸手术肺部并发症的危险因素，也是老年患者肺癌术后并发症的独立风险因素。术前至少戒烟4周。（2）保持口腔清洁：鼓励患者使用医用漱口水，每餐后漱口。保持口腔湿润，必要时进行口腔护理，每日1次-2次。（3）肺康复训练：①物理康复：主动呼吸训练（active cycle of breathing technique, ACBT）^[47]^，制定个性化且适宜的呼吸训练及运动训练计划，提高心肺耐受性，改善携氧量^[48]^。据研究^[49,50]^，ACBT可迅速清理老年患者呼吸道分泌物，延缓老年患者活动能力下降程度，清除痰液并训练肺功能，改善血氧饱和度，有利于术后肺功能的恢复，减少术后并发症的发生。老年肺癌术后患者采用基于阻力呼吸训练器的呼吸功能训练，能有效提高膈肌运动能力和肺功能，降低干预后并发症的发生率^[51]^。运动训练包括四肢运动：肢体的活动一方面能够维持正常的关节活动范围、肌肉的长度和肌力，另一方面通过活动改善肺容量、优化通气，术前运动能够帮助患者维持较好的心肺适能，提高手术的耐受能力。②体位管理：术后尽早尽快地采取直立位能够帮助患者改善通气、优化通气血流比，包括床上的翻身、坐起、体位转移、椅上坐起以及站立步行。③有效支撑伤口：尤其是术后咳嗽的伤口支撑，能有效缓解术后疼痛。如胸部行开放式手术的患者，术后咳嗽时可以将小的软枕抱于胸前。

**问题2：老年肺癌患者术后谵妄评估及预防**

**共识推荐：推荐使用意识模糊评估法（confusion assessment method, CAM）^[52]]^对患者进行评估，对高危患者做到早预防、早识别、早治疗，降低发病率。**

**证据：**谵妄是老年患者术后常见的不良反应^[53]^，发生谵妄的老年患者死亡风险增加1.5倍^[54]^，常发生于有认知障碍史的老年患者。

**观察护理：**建议使用老年人适用的评估方法CAM^[52]^对患者进行评估，提升护理人员对老年肺癌患者术后谵妄的识别能力，同时准确评估患者的疼痛程度，有效控制疼痛；夜间减少病房干扰，保证患者的睡眠；保证监护仪器的正常运行，提供充足的氧疗以改善缺氧；为视力障碍患者提供老花镜，为听力障碍患者提供助听器；保证大便通畅等。充分落实对高危患者的早预防、早识别、早治疗，以降低发病率。

**问题3：老年肺癌患者术后常见并发症的观察及护理**

**共识推荐：老年患者均有不同程度的心肺功能减退，术后应加强呼吸系统、循环系统相关并发症的观察及护理，即低氧血症、肺部感染、呼吸衰竭、肺水肿、心律失常、心肌梗死等；主要护理措施包括监测生命体征、积极协助排痰、充分吸氧、控制液体总量及输液速度、控制疼痛、保持水电解质及酸碱平衡、稳定患者情绪、做好饮食管理等。**

**（1）呼吸系统**

**证据：**老年肺癌患者对手术的创伤、失血、缺氧、麻醉等耐受性差，术后均出现机体代偿能力减退，心肺功能减弱，相关并发症的风险高。术后密切监护，观察心率、心律变化，应重点关注血氧饱和度监测以及血气分析结果，及早发现低氧血症。肺不张是肺感染的早期表现，肺感染多出现于术后48 h，由于老年肺癌患者体质差，肺泡表面的弹性减弱、支气管纤毛的排痰运动能力下降等原因，呼吸道内的分泌物不易排出，发生潴留，严重者会造成肺部感染。老年肺癌患者自身呼吸功能减退，术后有效呼吸面积减少、肺泡表面活性物质失去活性造成肺泡萎陷、组织间隙的液体回流障碍等情况，易造成肺间质水肿，影响肺组织的弥散功能，严重可造成呼吸衰竭^[55]^。部分老年肺癌患者心功能不全，心脏的储备能力弱，在手术创伤的外界刺激下会超出其代偿能力，易引发急性肺水肿。

**观察护理：**①低氧血症的护理：当患者发生痰鸣音加重、呼吸加快或血氧饱和度在85%-90%，提示分泌物阻塞呼吸道、痰液潴留或肺不张、肺部感染及胸腔积液等常见并发症，应急查血气分析及胸部X线检查。若血氧饱和度低于85%，提示成人呼吸窘迫综合征、呼吸衰竭的发生，应及时给予无创呼吸机和（或）气管切开治疗^[56]^。②肺部感染的护理：肺感染患者的临床表现：喘憋不能平卧、烦躁、心动过速、体温偏高、哮鸣音、面色发绀等症状。干预措施：鼓励患者练习深呼吸，间隔每2 h协助排痰，注意观察痰液的颜色、性质和量，痰液黏稠者全麻清醒后遵医嘱给予雾化吸入，可缓解气管痉挛及降低痰液黏稠性。咳痰无力者可按压喉部气管刺激反射性咳嗽，必要时可给予鼻导管吸痰，或者协助医生行床旁纤维支气管镜吸痰。关注体温，必要时进行痰培养，及时选用抗生素治疗^[57]^。③呼吸衰竭的护理：保证患者术后充分氧疗，保持呼吸道的通畅，防止痰液蓄积阻塞^[58]^，同时严密观察患者的呼吸节律，重点监测血氧和血气分析数据，必要时协助医生给予气管插管或呼吸机辅助通气，同时注意管路固定，防止脱出，有助于预防和减少呼吸衰竭的发生。④肺水肿的护理：保证持续有效的氧疗，控制术中和术后的输液量和输液速度^[59]^，加强听诊肺部有无细小水泡音或啰音，注意观察患者有无咳粉红色泡沫痰、意识不清、血压下降、呼吸困难及尿少等症状，出现以上症状时，高度怀疑肺水肿，应及时治疗和纠正导致肺水肿的病因，使用利尿剂，并进行呼吸支持。

**（2）循环系统**

**证据：**老年肺癌患者术后心律失常发生率较高^[60]^。术后诱发心律失常的主要危险因素是麻醉以及手术所致的缺氧，另外术后疼痛、电解质异常也是诱发因素。由于老年患者心脏功能逐渐退化^[61]^，冠状动脉粥样硬化发生率随增龄而增加，加上手术创伤，易导致冠状动脉缺血，引起心肌梗死^[62]^。

**观察护理：**（1）心律失常的护理：术后要鼓励咳嗽排痰，确保呼吸道通畅，充分氧疗，避免缺氧诱发心律失常。同时需安慰患者的紧张情绪，按医嘱合理应用止疼药。保持水电解质及酸碱平衡，尤其是血清钾水平。术后持续监测心电图的变化，一旦发现应及时处理，降低因心律失常恶化导致心搏骤停甚至死亡的风险。（2）心肌梗死的护理：老年患者护理应注意保持情绪稳定，忌过分激动和悲伤；早期活动以促进胃肠功能恢复，合并便秘者使用通便药物，保持大便通畅；饮食遵循少量多餐原则，清淡易消化，忌过饱、过快进食；控制室温适宜，过热过冷均容易诱发心律失常，进一步造成心肌梗死。

## 老年肺癌患者放疗期间护理

**问题1：老年肺癌患者放射性肺炎的评估及观察护理重点**

**共识推荐：老年肺癌放疗后1个月-3个月应重点监测患者发热、刺激性咳嗽、咳痰、心悸、胸痛等症状，应动态监测患者的生命体征，对其呼吸功能进行全面评估，根据患者存在的症状及评估结果进行对症的观察及护理。放射性肺炎的治疗常用糖皮质激素联合抗生素，并辅以平喘、化痰、止咳等药物，应做好用药指导。**

**证据：**放射性肺炎是肺癌患者胸部放疗最主要的并发症，通常发生于放疗后1个月-3个月^[63]^。主要表现为发热、刺激性干咳，伴咳痰、气紧、胸痛和心悸，偶有高热。轻症患者可无明显症状，肺部炎症可自行消散。重症患者可出现广泛肺纤维化和肺部实变，气紧症状和肺部感染随肺纤维化程度加重而进行性加剧，严重时出现呼吸衰竭和咯血。老年患者肺功能减退，尤其合并其他慢性疾病的老年患者，身体状况较差，发生放射性肺炎的风险较高。

**观察护理：**动态监测生命体征的变化，对其呼吸功能进行全面评估。观察患者的呼吸频率、深度与节律以及有无气促及呼吸困难；观察有无咳嗽、咳痰、咯血量以及其频率，观察患者的血氧饱和度以及皮肤、甲床颜色的变化。放射性肺炎患者的病情变化快，护理人员对患者应加强巡视，对此类患者的照护需更细致。

**问题2：老年肺癌患者放射性食管炎的评估及观察护理重点**

**共识推荐：放疗前评估患者年龄、合并基础疾病（高血压、糖尿病^[64]^）、同期化疗、平均照射剂量、放疗时间、临床靶区的大小等相关因素，综合评估可能发生放射性食管炎的风险。对于低风险的老年肺癌患者，若合并糖尿病或高血压等基础疾病，首先应给予降血糖或降压药物，减少基础疾病对其放射治疗后的影响，护理人员需定期检查老年肺癌患者进食情况，忌辛辣、烟酒等刺激性食物，鼓励适量多饮水，清淡饮食，注意口腔清洁，避免继发性食管炎的发生；对于中、高风险的老年肺癌患者，口服复合维生素B，增加检查食管的频次，一旦发现食管异常情况，立即做好相应护理干预，可有效降低放射性食管炎的发生率^[65]^。**

**证据：**放射性食管炎常于放疗结束后1周至数周内发生，主要症状为吞咽疼痛、吞咽梗阻、胸骨后疼痛、食道烧灼感。评估患者的病情及全身状况，密切观察生命体征、疼痛及呼吸状况，了解患者有无饮水呛咳情况。特别是老年患者更要高度重视。

**观察护理：**放射性食管炎患者进食应少量多餐，不宜过饱，进食后勿马上平卧^[66]^。患者进餐前后可适量饮温水以冲洗食管，并保持口腔清洁，每天饮水量2,000 mL以上。食管吞咽疼痛的患者，可餐前30 min遵医嘱吞服小磨香油10 mL联合阿莫西林0.5 mg及云南白药0.5 mg^[58]^，每日少量多次口服用药，并嘱患者服用时先含在口内，平躺后再缓慢下咽，去枕平卧30 min，使药物与食管黏膜表面长时间接触。当出现进食梗阻、胸部剧痛、呼吸困难、呕吐、呕血等症状时，提示可能发生了食管穿孔或食管气管瘘。一旦确诊，应立即禁食禁饮，暂停放疗，给予对症支持治疗，准确记录出入量，遵医嘱补充足量液体和电解质。研究发现，中药对放射性食管炎的症状改善具有一定治疗价值。刘俊德等研究^[67]^显示，沙参麦冬汤能调节放射性食管炎患者血清中炎性因子的表达，改善人体免疫功能。也有研究报道，养阴清热解毒方^[68]^和清热养阴利咽方^[69]^可有效预防放射性食管炎的发生，减轻患者炎性反应症状。指导患者遵医嘱使用药物，并注意观察药物作用与不良反应。

**问题3：老年肺癌患者放射性皮炎的评估及观察护理重点**

**共识推荐：放疗期间，护理人员每天观察评估患者放射野皮肤有无放射性皮肤损伤发生及皮肤损伤的程度，指导患者正确保护放射野皮肤不受其他不良刺激。**

**证据：**放疗会导致患者出现不同程度的皮肤反应^[70]^。放射性皮肤反应包括干性反应和湿性反应。干性皮肤反应主要表现为皮肤干燥、瘙痒、色素沉着、红斑及脱皮，没有渗出物，造成感染的概率不大，但能产生永久性浅褐色斑。湿性皮肤反应是指照射区皮肤有湿疹、水泡，甚至出现破溃、糜烂，常常发生在皮肤多汗、褶皱处，如颈部、腋下等部位。老年肺癌患者皮肤干燥、松弛，很容易发生放射性损伤。

**观察护理：**放疗期间指导老年肺癌患者正确使用皮肤保护剂。轻度的放射性皮炎不需要治疗，放疗结束后即可慢慢自行恢复。比较严重的放射性皮炎可以通过局部涂抹激素类药膏或者激素+抗生素+中药膏联合治疗。放射野内的皮肤注意加强护理，衣物要柔软，减少摩擦以免加重皮肤损伤。发生放射性皮炎的患者要注意局部皮肤清洁，勿用刺激性清洁用品，加强暴露，保持干燥。医用射线防护剂可有效治疗放疗导致的急性皮肤反应，还能延缓皮肤损伤的发生^[66]^。Primavera等^[71]^研究表明，透明质酸可降低放射性皮炎的严重程度。局部糖皮质激素^[72]^、金盏菊制剂^[73]^以及磺胺嘧啶银乳膏^[74]^对放射性皮炎具有一定的预防作用。也有研究表明，芦荟凝胶^[75]^、烧伤膏^[76]^、银敷料^[77]^对放射性皮炎均无预防作用。Meta分析结果^[78]^显示，三乙醇胺不能用于预防放射性皮炎，也不能降低放射性皮炎的发生率。

**问题4：老年肺癌合并脑转移行脑部放疗患者放射性脑损伤的评估及观察护理重点**

**共识推荐：放疗结束后6个月-3年，重点监测老年肺癌患者脑功能减退、身体灵活性减慢等情况，如身体机能改变，部分患者存在步态不稳、智力减退、言语不清、认知障碍、记忆力下降等脑损伤表现。根据患者存在的症状及评估结果进行对症观察及护理。**

**证据：**肺癌患者伴脑转移，常需行脑部放疗。放疗后部分患者会出现神经细胞和颅内血管受损的病理生理改变以及脑部影像学上的变化。这种放射性脑损伤可发生在放疗后的任何时间，以照射结束后6个月-3年最常见。老年肺癌患者脑功能逐渐减退，身体灵活性减慢，放疗后更容易出现身体机能改变，部分患者存在步态不稳、言语不清、智力减退、记忆力下降等脑损伤表现，护理人员应全面、仔细地评估，耐心询问患者的需求。

**观察护理：**（1）灵性照顾：放射性脑损伤患者的病程长、预后差，患者及家属心理压力大，部分患者自杀风险高。护理人员需正确评价患者的灵性问题，并采取针对性的个体化护理措施。（2）生活护理：护理人员应耐心询问患者的需求，并严密监测患者的日常活动，必要时要求家属24 h陪护。保持病房、走廊、卫生间地面干净整洁，加强跌倒、坠床的风险提示及相关设施、设备的安装，预防安全不良事件的发生。脊髓损伤导致膀胱括约肌失调，患者需长时间留置尿管，每天应进行会阴部护理，每周更换尿袋2次，每2周更换尿管1次，必要时用呋喃西林进行膀胱冲洗，严控尿路感染^[79]^。偏瘫患者应保持床单元整洁、干燥，每1 h-2 h翻身、拍背一次，预防坠积性肺炎和压力性皮肤损伤。（3）呕吐的护理：呕吐是放射性脑损伤的常见症状。这类患者应密切监测生命体征、意识及瞳孔的变化，警惕脑疝的发生。抽搐患者根据病情适时进行约束、垫牙垫，防止意外伤害，必要时使用镇静剂，颅内压增高者应采取降颅内压措施^[80]^。（4）呼吸道的护理：放射性脑损伤患者呼吸道分泌物较多时易引起误吸，进而导致呼吸道阻塞和肺部感染，及时清理呼吸道是抢救颅脑损伤患者的关键。保持呼吸道通畅、及时有效地吸痰是放射性脑损伤患者的重要护理措施。在吸痰操作前、中、后应采用高流量吸氧，预防脑缺血缺氧、颅内压升高，以免加重脑水肿^[81]^。患者呕吐时勿憋气，气管切开患者适时吸痰，以防窒息。气管插管的患者应严格执行无菌操作，做好呼吸道的护理，保证气道的湿化。（5）用药护理：放射性脑损伤患者首选甘露醇降低颅内压。甘露醇具有一定肾脏毒性，对静脉血管有较大的刺激性。采用甘露醇脱水治疗时，护理人员应记录24 h尿量，观察尿液颜色、性质、量，适时监测尿素氮和血肌酐^[82]^。早发现、早诊断、早治疗肾功能损伤，去除危险因素，促进肾功能恢复。同时，选择深静脉输入，保护血管。糖皮质激素是治疗放射性脑损伤引起的炎症反应的重要方法之一。但糖皮质激素可导致消化道溃疡，护理人员需监测患者生命体征，注意观察排便次数、性状及量，及时留取大便标本送检，以明确有无隐血情况发生。消化道一旦大出血可造成失血性休克，甚至危及生命，应及时监测生命体征及病情的变化。可以在神经内科医师指导下应用营养神经、改善神经功能的药物。

## 老年肺癌患者化疗期间的护理

**问题1：老年肺癌患者输注化疗药物静脉通路管理有哪些?**

**共识推荐：推荐根据患者自身情况（自理能力、合作程度、经济条件、既往史等）、化疗方案、血管条件、置管禁忌证、患者的舒适度等选择合适的静脉通路。老年肺癌患者的静脉血管壁增厚，弹性降低，脆性增加，输注刺激性强的化疗药物应首选经外周静脉置入的中心静脉导管（peripherally inserted central catheter, PICC）。在满足治疗需要的情况下，选择直径最小和长度最短的导管进行治疗^[83]^；由于PICC需长期留置且有外露导管，相关并发症发生率较其他中心静脉置管要高，故应重视对老年PICC置管患者认知功能的评估，对于认知功能障碍的患者采取针对性的健康教育措施，同时可将患者家属纳入健康教育中，并做好PICC相关并发症的预防和处理。**

**证据：**PICC常见并发症有感染、血栓、静脉炎、导管脱出等。老年人因其生理特点（血液黏稠、免疫力低下等）、认知功能障碍（患者不能正确执行活动注意事项，在导管维护的频率、异常症状的识别及处理方式等方面记忆和辨识能力减弱，缺乏主动增加饮水量和肢体活动的意识等^[84]^）是导管相关血流感染、医用黏胶相关性皮肤损伤（medical adhesive related skin injury, MARSI）和血栓的好发人群。

**观察护理：**（1）认知功能评估及护理：轻度认知功能障碍的老年患者，常常症状不明显，且不影响日常生活，特别容易被忽视。护理人员需重视对老年PICC置管患者认知功能的评估，及时采取个性化的健康教育，同时鼓励患者家属参与到健康教育中。（2）导管相关血流感染的预防：导管置入、使用、维护等均严格执行无菌操作；每日评估导管保留必要性；及时拔除不必要的导管。（3）导管相关血栓的预防及措施：选用合适的导管型号，导管的直径与血管腔的比值小于45%；提高一次性穿刺成功率；饮水量在日常的基础上适当增加，避免诱发心、脑血管事件；置管肢体应每日进行血栓预防活动，如做血栓预防操，每日3次，每次20个-30个握拳运动；置管24 h后热敷置管部位肢体，每日3次，每次20 min-30 min，以促进血液循环^[85]^。当置管肢体感觉肿胀疼痛、臂围/腿围增大超过3 cm时，应进行静脉血管彩超筛查血栓^[86]^。患者带管期间，应向患者及其家属进行静脉通路维护的相关宣教，特别是PICC。宣教内容包括带管出院应定期维护、着宽松的衣物、置管肢体避免受压、置管肢体应适当活动；教会患者自我观察（穿刺点有无红肿热痛、敷料松脱、卷边、导管脱出等），并及时通知医护人员处理。（4）MARSI：MARSI常发生在肿瘤患者PICC置入部位，常见机械性损伤和接触性皮炎，主要危险因素包括频繁更换透明敷料、穿刺肱二头肌内侧、使用药物紫杉醇等。护理人员在管路维护过程中需重点关注以上危险因素，做到防治并举，加强具有高危因素患者的宣教，加强患者对自身PICC置入部位皮肤的管理，提高其自我护理的依从性。护理人员在黏胶剂的选择、粘贴的力度及手法、揭除的技术方面应进行规范。

**问题2：老年肺癌患者化疗相关的恶心呕吐（chemotherapy induced nausea and vomiting, CINV）的评估及观察护理**

**共识推荐：推荐使用Dranitsaris的CINV风险评分系统对恶心呕吐症状进行评估，严格按时正确给药，及时评估止吐效果。适时向医生报告患者用药后的止吐效果，以及时调整止吐方案。**

**证据：**CINV是肿瘤化学治疗期间常见的不良反应之一，有研究^[87,88]^认为，如果不加以干预或仅使用常规止吐剂干预，患者恶心呕吐的发生率仍高达54%-96%，CINV不仅会降低老年肺癌患者的生活质量，还会导致患者的依从性降低，严重呕吐会造成机体的水电解质失衡和营养不良^[88]^，影响治疗效果，使肿瘤得不到有效的控制。因此老年肺癌患者支持治疗的重要内容包括预防或减少CINV的发生^[89]^。

**观察护理：**（1）合理安排化疗用药时间：CINV根据发生的时间可分为急性和延迟性，其中急性CINV通常在化疗后几分钟至几小时内发生。静脉化疗药物宜在胃内容物排空后使用，此时胃内充盈小，出现呕吐症状少。正常胃内容物排空时间为2 h-4 h，而老年人餐后胃蠕动和收缩力降低，胃排空延迟，故老年人可适当延后。（2）准确记录24 h出入量： 密切观察呕吐物的颜色、性质、量，及时清洁口腔并处理呕吐物，避免对患者的持续刺激。监测患者体重、水电解质情况。必要时根据患者情况补液，保证水、电解质平衡。（3）饮食指导：保证良好的用餐环境，适当通风，避免进餐时的不良刺激，如异味、污物等。老年患者的味觉、咀嚼及消化功能等均下降，应进行个体化的饮食指导。食物应选择高蛋白、高热量、富含维生素、易消化的低脂软食，少量多餐。（4）非药物干预：在使用药物干预的同时，可结合适当的非药物干预来提高止吐的效果，例如：音乐疗法、放松疗法、睡眠诱导等^[90,91]^。

**问题3：老年肺癌患者化疗导致的便秘及其预防和治疗**

**共识推荐：推荐老年肺癌患者化疗期间采用饮食结构调整、行为方式调整、物理干预、药物治疗等方式进行预防及治疗。**

**证据：**便秘是老年人的常见症状，约占老年人群的1/3。同时治疗CINV的5-羟色胺3受体拮抗剂可抑制胃肠道分泌及蠕动功能^[92]^，导致老年化疗患者出现便秘的风险更高。便秘的发生会延长粪便在患者体内的滞留时间，产生大量毒素，影响治疗，同时降低患者的舒适感。

**观察护理：**（1）饮食调整：嘱患者食用高纤维素的食物，如豆芽、红薯、南瓜等，刺激肠蠕动；饮水量在日常的基础上适当增加，避免诱发心、脑血管事件^[93]^。（2）行为方式调整：根据患者个体情况制定相应的锻炼计划，如散步、打太极拳等。每日建议进行30 min-60 min的活动，促进肠道蠕动，卧床患者可通过挥臂、转体等方式锻炼。同时应定时排便，排便时不宜携带手机、书籍等分散注意力的物品，建立良好的习惯。（3）物理干预：腹部的环形按摩、盆底肌的训练等，可加快肠道的蠕动，增加肠道平滑肌肌张力，达到改善肠道功能的目的。（4）饮食行为调整无效者：遵医嘱给予缓泻剂和软化剂，观察并记录患者的排便量、排便时间、性质及颜色等。

**问题4：老年肺癌患者化疗后导致的骨髓抑制的评估及观察护理**

**共识推荐：化疗后骨髓抑制的评定标准，专家推荐使用世界卫生组织制定并公布的血液学分度标准^[94]^。主要危险因素包括存在营养风险、化疗前血红蛋白水平偏低、铂类化疗药物、合并两种或以上其他不良反应等^[95]^。针对老年肺癌化疗患者，护理人员应关注以上危险因素，及时评估风险，做好感染预防性护理的指导，加强营养摄入，密切监测化疗前后血象变化（血常规及肝肾功能等），做到及早发现、及时干预，改善生活质量。**

**证据：**存在营养不良问题的老年肺癌患者，因营养不良引起的白蛋白降低更易发生骨髓抑制；化疗前若血红蛋白水平降低，提示老年肺癌患者的造血功能减弱，也会进一步增加骨髓抑制的发生风险^[96,97]^；铂类常会减少周围血细胞数量，加重细胞的毒性作用，造成骨髓抑制；当合并两种或以上化疗不良反应时，提示患者对化疗耐受性降低，在同等剂量化疗药物的情况下更容易发生骨髓抑制^[98]^。

**观察护理：**（1）白细胞水平偏低的护理：白细胞特别是中性粒细胞水平下降时，患者发生感染的风险增加。此时应保持室内空气流通，加强个人卫生，严密监测体温。增加病房消毒次数，严格无菌操作，减少探视。当白细胞<1×10^9^/L，患者应佩戴口罩，并进行保护性隔离^[99]^。（2）血小板水平偏低的护理：血小板水平偏低时，应指导患者进食软食，避免大便干燥，避免剔牙、抠鼻等动作。每日观察并教会患者观察全身皮肤/黏膜有无出血点、大便颜色等。血小板水平低于50×10^9^/L时，嘱患者卧床休息。护理人员应协助老年肺癌患者做好生活护理，减少活动，避免磕碰。当血小板水平低于10×10^9^/L时，患者发生自发性出血的风险极高^[100]^，应绝对卧床休息，若出现头痛、恶心呕吐等症状时应及时通知医生给予处理。

**问题5：老年肺癌患者癌因性疲乏（cancer-related fatigue, CRF）评估及观察护理**

**共识推荐：推荐使用简易疲乏量表（brief fatigue inventory, BFI）、癌症疲乏量表（cancer fatigue scale, CFS）等评估量表进行评估；需要多次评估时则可采用数字评分尺（0分-10分）等简易工具进行评估^[101]^。在评估的基础上对患者进行健康教育、运动干预、心理社会支持。**

**证据：**美国国立综合癌症网络将CRF定义为一种与癌症或癌症治疗有关的痛苦、持续、主观的疲劳感或疲惫感^[102]^。超过半数的化疗患者长期存在CRF的症状。老年人由于机体储备能力下降，在化疗期间更容易受到CRF的困扰。

**观察护理：**（1）健康教育：护理人员应在正确评估的基础上，对患者及其主要照顾者进行CRF健康宣教，包括CRF的评估方法、影响因素^[103]^。对于接受化疗的患者，应强调CRF是常见症状，并不是因为病情加重或治疗无效；良好的健康教育能够减轻化疗患者的疲乏程度。（2）运动干预：运动能够调节大脑内啡肽的分泌以缓解患者疲乏症状，并提高癌症患者的血红蛋白水平及心肺功能，国内外的研究^[104,105]^都证实适当运动能够缓解CRF。在进行运动干预时，需要结合老年患者的自身情况，明确活动强度、时长和频率，制定个体化的运动计划。适宜老年患者的锻炼项目有散步、慢跑、游泳、跳舞、太极拳等。同时，正在接受化疗的患者对运动干预的依从性较差，护理人员可通过专业指导、定期评估和训练同伴等方式提高患者依从性。（3）心理社会支持：CRF给患者带来的影响是多维度的，包括生理、心理和社会等方面，容易存在焦虑、抑郁等情绪。护理人员可通过认知行为疗法、心理教育疗法、表达支持疗法等^[106,107]^方式，帮助患者重拾战胜疾病的信念和积极的应对方法；适时提供情感支持，加强家人的陪伴。

## 老年肺癌患者靶向治疗和免疫治疗期间护理

**问题1：老年肺癌患者行靶向治疗和免疫治疗期间腹泻症状的评估及观察护理**

**共识推荐：针对消化道不良反应，美国国立癌症研究所（National Cancer Institute, NCI）制定了不良事件通用术语标准（Common Terminology Criteria for Adverse Event, CTCAE）5.0分级标准，除以上分级外，还需对以下内容进行评估：（1）准确评估发生腹泻的时间；（2）准确记录大便的频次、颜色、性状和量；（3）若出现发烧、晕眩等症状，警惕可能伴随其他更严重的不良反应；（4）根据评估患者的饮食特点和治疗的依从性来进行饮食指导及补液治疗，并做好肛周皮肤的管理。**

**证据：**腹泻是指大便次数明显增多和大便性状发生改变^[108]^。严重腹泻者可出现脱水症状，即主诉口渴明显、皮肤黏膜弹性变差等，少数甚至伴有意识改变、面色苍白、外周白细胞水平明显增高、高热或体温不升等中毒表现^[108]^。

**观察护理：**护理人员需严密观察患者腹泻次数、颜色、性质及量。重点关注肛周的皮肤情况，无异常的情况下使用温水擦拭，若肛周皮肤红肿，建议使用氧化锌软膏或红霉素软膏外涂。鼓励患者在治疗期间宜少食多餐，清淡低脂低纤维饮食，避免油腻食物，忌食咖啡因、酒精、奶制品、葡萄汁以及辛辣食物。注意保护患者安全，预防跌倒，治疗期间留专人陪伴。若2级腹泻且持续时间>48 h，注意观察有无脱水或电解质失衡，一旦发生应及时输液治疗，每日饮用等渗液体量1 L-1.5 L，可使用洛哌丁胺、益生菌和思密达等予以治疗^[109]^。若患者出现3级及以上腹泻，需住院监测，行粪便显微镜检查；每天饮用1 L-1.5 L等渗液体，静脉给予补充治疗至少24 h，酌情考虑给予预防性抗感染及生长抑素治疗，必要时停用表皮生长因子受体酪氨酸激酶抑制剂（epidermal growth factor receptor-tyrosine kinase inhibitors, EGFR-TKIs）治疗。

**问题2：老年肺癌患者靶向治疗和免疫治疗期间皮疹及甲沟炎的评估及观察护理**

**共识推荐：推荐参考2017年NCI发布的CTCAE 5.0标准进行分级^[110]^。根据评估结果进行健康指导及药物干预。**

**证据：**患者在靶向药物EGFR-TKIs治疗后1周-2周^[111]^常出现皮疹/痤疮样皮疹，发生于面、胸、上背等皮脂腺丰富的人体部位，严重时下肢甚至全身均会出现；皮疹形态单一，以丘疹脓疱疹为主，皮肤干燥且多伴瘙痒，这种不适感会导致老年肺癌患者心烦意乱，影响休息及睡眠^[112]^。

**观察护理：**（1）皮疹护理及治疗要点：①加强健康宣教，在使用EGFR-TKIs治疗前，医护人员应告知患者皮疹是靶向治疗常见的不良反应，皮疹的症状越重，提示疗效越好，EGFR-TKIs所致的皮疹不具有传染性；②严密观察患者的皮肤状况，是否出现皮疹、瘙痒、干燥、龟裂等情况，一旦发现及时处理；③指导有效的预防措施，如嘱咐防晒，推荐使用广谱防晒品（防晒系数≥30）；④加强皮肤护理，保持皮肤清洁的同时还需保持湿润，使用温水洗澡，且沐浴后涂抹保湿乳剂，若皮肤瘙痒，建议使用棉柔毛巾，避免使用碱性肥皂，禁止挠抓；⑤EGFR-TKIs治疗过程中嘱咐患者穿着柔软的衣物以减少皮疹部位的摩擦，选择透气性好且宽松的鞋子，每次温水沐足后涂抹润肤霜，不仅可有效预防足部皮疹，而且可治疗足癣；⑥针对1级-2级痤疮样皮疹，予以炉甘石洗剂以及喜辽妥软膏外敷治疗即可，伴有症状者可加用2.5%氢化可的松霜剂、抗生素或抗过敏药；针对3级-4级皮疹患者，根据情况可进行细菌/真菌/病毒培养，治疗方法是口服抗生素，同时糖皮质激素局部外涂，对于顽固性瘙痒患者，可考虑使用普瑞巴林或加巴喷丁等药物，但若效果不佳，需及时停止靶向药物，待皮疹消退。（2）甲沟炎护理及治疗要点：①加强皮肤护理，保持手部和足部皮肤清洁干燥，不要将手和脚浸泡在肥皂水中，经常使用滋润霜；②为预防甲沟炎发生，修剪指甲时要小心，避免指甲受伤，注意预防甲下血肿的形成，可局部使用类固醇激素预防红斑扩大；③指导患者穿宽松、舒适的鞋子保护趾甲，穿鞋前确保脚部干燥，避免皮肤受刺激；④针对1级-2级甲沟炎，指导患者外用抗生素（克林霉素、夫西地酸、百多邦）及白醋浸泡（手浸泡于含1:1白醋与水的混合液中，每天15 min）^[113]^，必要时还需外用强效的糖皮质激素和抗生素/抗真菌药物；⑤针对3级及以上的甲沟炎者，依据说明书来调整药物剂量，必要时根据情况可行细菌/真菌/病毒培养，口服多西环素等抗生素治疗，必要时予以拔甲。

**问题3：老年肺癌患者免疫治疗期间相关肺炎护理重点**

**共识推荐：免疫相关性肺炎患者的病情变化快，需对其各项生命体征及临床症状包括低热、刺激性干咳、咳痰、气紧、胸痛和呼吸困难、高热等进行动态监测及对症治疗及护理。应加强巡视，确保患者静脉输液通畅，并注意输液速度。**

**证据：**免疫相关性肺炎为不常见的不良反应，但却是严重的不良反应^[114]^。免疫相关性肺炎主要表现为低热、刺激性干咳，伴咳痰、气紧、胸痛和呼吸困难，偶有高热。少数患者虽没有临床症状，但肺部影像学变化明显。肿瘤患者接受抗细胞毒T淋巴细胞相关抗原4（cytotoxic T lymphocyte-associated antigen-4, CTLA-4）单抗治疗发生肺炎的患者较少，程序性死亡受体1（programmed cell death 1, PD-1）/程序性死亡配体1（programmed cell death ligand 1, PD-L1）单抗或联合治疗的患者发生肺炎的概率更高^[115]^。

**观察护理：**（1）发热护理：免疫相关性肺炎的主要临床症状之一为发热，对于高热患者，需及时采取物理或药物降温措施。根据出汗量为患者更换衣物和床褥，预防压力性损伤，并注意患者的保暖。发热患者还应加强营养补充，饭后刷牙，保持口腔清洁卫生。（2）呼吸困难的护理：呼吸困难的患者宜选择半卧位以保持呼吸通畅，并持续吸氧。也可指导患者采用缩唇式呼吸。如出现急性呼吸困难，应使其保持呼吸道畅通，增加氧流量，并通知医生进行抢救。必要时，还应安置无创或有创呼吸机辅助通气。（3）用药护理：免疫相关性肺炎常用糖皮质激素联合抗生素，并辅以氧气吸入、镇咳化痰平喘等对症治疗。老年免疫相关性肺炎患者应尽早、足量、足疗程使用糖皮质激素及抗生素治疗，防止肺部感染加重，并遵医嘱使用祛痰药和支气管扩张剂，必要时吸氧。在用药过程中，护理人员应注意观察药物的不良反应，尤其是长期、大剂量使用糖皮质激素的老年患者，可导致真菌感染以及激素减量过程中病情反复。临床症状明显好转后糖皮质激素应逐渐减量至停用。

**Table:** 

参与本共识的专家组成员	
执笔专家	
李梅	天津医科大学总医院
陈军	天津医科大学总医院， 天津市肺癌研究所
杨梅	四川大学华西医院
纪慧慧	天津医科大学总医院
余春华	四川大学华西医院
石怡	天津医科大学总医院
樊榕榕	北京大学人民医院
邢颖珺	天津医科大学总医院
郭玲玉	天津医科大学总医院
牟倩倩	四川大学华西医院
郑儒君	四川大学华西医院
李俊英	四川大学华西医院
陈林	四川大学华西医院
陶诗琪	四川大学华西医院
参加撰写专家	
王鑫	天津医科大学总医院
赵荣志	天津医科大学总医院
张琳琳	天津医科大学总医院
徐嵩	天津医科大学总医院
李昕	天津医科大学总医院
赵洪林	天津医科大学总医院
赵青春	天津医科大学总医院
董明	天津医科大学总医院
